# Splanchnic Vein Thrombosis in Inflammatory Bowel Disease: An Observational Study from the ENEIDA Registry and Systematic Review

**DOI:** 10.3390/jcm12237366

**Published:** 2023-11-28

**Authors:** Maria Puig, Helena Masnou, Francisco Mesonero, Luís Menchén, Luís Bujanda, Jesús Castro, Irene González-Partida, Raquel Vicente, Carlos González-Muñoza, Marisa Iborra, Mónica Sierra, José María Huguet, María José García, Ruth De Francisco, Francisco Javier García-Alonso, Míriam Mañosa, Eugeni Domènech, on behalf of ENEIDA-GETECCU Registry

**Affiliations:** 1 Gastroenterology & Hepatology Department, Hospital Universitari Germans Trias i Pujol, 08916 Badalona, Spain; mariapugo93@gmail.com (M.P.); mmanosa.germanstrias@gencat.cat (M.M.); eugenidomenech@gmail.com (E.D.); 2 Gastroenterology Department, Hospital Universitario Ramón y Cajal, 28034 Madrid, Spain; pacomeso@hotmail.com; 3 Centro de Investigación Biomédica en Red de Enfermedades Hepáticas y Digestivas (Ciberehd), 28222 Madrid, Spain; luisalberto.menchen@salud.madrid.org (L.M.); luis.bujandafernandezdepierola@osakidetza.eus (L.B.); 4 Gastroenterology Department, Instituto de Investigación Sanitaria Gregorio Marañón, Hospital General Universitario, 28007 Madrid, Spain; 5 Gastroenterology Department, Biodonostia Health Research Institute, Universidad del País Vasco (UPV/EHU), 20018 San Sebastián, Spain; 6 Gastroenterology Department, Hospital Clínic i Provincial, IDIBAPS, 08036 Barcelona, Spain; jecastro@clinic.cat; 7 Gastroenterology Department, Hospital Puerta de Hierro, 28222 Madrid, Spain; irenegonzalezpartida@gmail.com; 8 Gastroenterology Department, Hospital Universitario Miguel Servet, 50009 Zaragoza, Spain; 9 Gastroenterology Department, Hospital de Santa Creu i Sant Pau, 08025 Barcelona, Spain; cgonzalezm@santpau.cat; 10 Gastroenterology Department, Hospital Politècnic La Fe, 46026 Valencia, Spain; 11 Complejo Asistencial Universitario de León, 24008 León, Spain; msierraau@saludcastillayleon.es; 12 Hospital General Univeristari de València, 46014 Valencia, Spain; josemahuguet@hotmail.com; 13 Hospital Universitario Marqués de Valdecilla e IDIVAL, Universidad de Cantabria, 39005 Santander, Spain; garcia_maria86@hotmail.com; 14 Hospital Universitario Central de Asturias, Instituto de Investigación Sanitaria del Principado de Asturias (ISPA), 33011 Oviedo, Spain; ruthdefrancisco@hotmail.com; 15 Hospital Río Hortega, 47012 Valladolid, Spain; fj.garcia.alonso@gmail.com; 16 Departament de Medicina, Universitat Autònoma de Barcelona, 08193 Bellaterra, Spain

**Keywords:** prevalence, splanchnic vein thrombosis, inflammatory bowel disease, outcome

## Abstract

Background: Thromboembolic events are frequent among patients with inflammatory bowel disease (IBD). However, there is little information on the prevalence, features and outcomes of splanchnic vein thrombosis (SVT) in patients with IBD. Aims: To describe the clinical features and outcomes of SVT in patients with IBD and to perform a systematic review of these data with published cases and series. Methods: A retrospective observational study from the Spanish nationwide ENEIDA registry was performed. A systematic search of the literature was performed to identify studies with at least one case of SVT in IBD patients. Results: A new cohort of 49 episodes of SVT from the Eneida registry and 318 IBD patients with IBD identified from the literature review (sixty studies: two multicentre, six single-centre and fifty-two case reports or case series) were analysed. There was a mild predominance of Crohn’s disease and the most frequent clinical presentation was abdominal pain with or without fever followed by the incidental finding in cross-sectional imaging techniques. The most frequent SVT location was the main portal trunk in two-thirds of the cases, followed by the superior mesenteric vein. Anticoagulation therapy was prescribed in almost 90% of the cases, with a high rate of radiologic resolution of SVT. Thrombophilic conditions other than IBD itself were found in at least one-fifth of patients. Conclusions: SVT seems to be a rare (or underdiagnosed) complication in IBD patients. SVT is mostly associated with disease activity and evolves suitably when anticoagulation therapy is started.

## 1. Introduction

Splanchnic vein thrombosis (SVT), which refers to the thrombosis of the portal vein, its intrahepatic branches, and the mesenteric, splenic or hepatic veins, is a rare condition in non-cirrhotic patients. Its reported incidence and prevalence depend heavily on the design of the study [1,2], though it is likely to be underestimated as its diagnosis is often incidental.

Several studies have reported an increased risk of venous thromboembolism among inflammatory bowel disease (IBD) patients, together with an increased associated mortality rate [3,4,5,6]. The reported incidence of thromboembolic complications among IBD patients ranges from 1% to 8% [7], increasing to 40% in autopsy studies [8]. The pathogenesis of thrombosis in IBD is not well established and a multifactorial mechanism has been suggested, though inflammatory disease activity seems to be the most important factor [7,9,10]. The clinical presentation of SVT is usually nonspecific; it may be confused with IBD flares or complications and may even run its course asymptomatically. This is the reason why some authors suggest that SVT may frequently be underdiagnosed [4,11]. Globally, in 50 to 70% of cases of SVT, there are co-existing systemic risk factors, including inherited thrombophilia and acquired prothrombotic conditions, while in 20–30% of cases, there is a local risk factor, such as abdominal surgery, infection or trauma, among others [2]; however, this issue has been scarcely assessed in IBD.

Limited information is available on the epidemiology and clinical outcomes of SVT among IBD patients. The reported prevalence of SVT in IBD ranges from 0.11 to 1.7% in non-selected cohort series [5,9,12,13,14,15,16] up to 10.8% among IBD patients with thrombotic events [15] or 45% among IBD patients undergoing an abdominal CT scan [17]. However, most studies addressing SVT in IBD are small-sized and some of them are restricted to portal vein thrombosis, which limit the possibilities of acquiring knowledge of the associated risk factors and its natural history and leads to a lack of recommendations for its evaluation and treatment. Given that SVT may be the origin of portal hypertension and it can also be the first manifestation of prothrombotic conditions, it would be of interest to expand the current knowledge on it. Therefore, the main objective of this study was to describe the clinical presentation, management and outcomes of SVT on all available published data and to include a new, large, multicentric, retrospective series of SVT in IBD patients from the ENEIDA registry in this systematic review. 

## 2. Patients and Methods

### 2.1. Patients

All adult patients included in the Spanish nationwide registry of IBD patients (ENEIDA) [18] who were also diagnosed with SVT, defined as a thrombosis involving the main trunk of the portal vein and/or its intrahepatic branches, suprahepatic, superior mesenteric, inferior mesenteric or splenic veins, were identified. Patients with thrombotic events of unspecified locations in the registry were also identified. Only patients with confirmed SVT and detailed information available on the episode of SVT after a careful medical record review were included. For the purpose of our study, the following variables were mandatory: date of SVT, findings in the diagnostic imaging technique, type and duration of SVT-related treatment, and disease activity and treatment of IBD at the time of SVT. 

The ENEIDA registry was approved by the Ethics Committee of all the participating centres, and patients gave their signed informed consent.

### 2.2. Data Collection

Among the collected variables, we included epidemiological and demographic characteristics, relevant comorbidities (haematological and immunological disorders, previous malignancies and surgeries, history of thrombotic events, pregnancy and miscarriage) and IBD characteristics (disease phenotype according to the Montreal classification, disease duration, previous surgeries and associated complications), as well as the clinical features of the IBD at the time of SVT diagnosis (disease activity according to the partial Mayo score for ulcerative colitis or the Harvey–Bradshaw index for Crohn’s disease, intraabdominal complications, treatments, and laboratory parameters). Regarding SVT, clinical and radiological features, the extent and aetiological work-up of SVT (including haematological, immunological and neoplastic disorders, and the existence of local inflammatory conditions) and treatment were collected. Finally, the resolution of thrombosis and the development of portal hypertension-related complications during follow-up were also collected when available. 

### 2.3. Statistical Analysis

Categorical variables are expressed as absolute values and frequencies and continuous variables are expressed as the median and interquartile range (IQR). All statistical analyses were carried out with the SPSS statistical package version 23.

### 2.4. Systematic Review

Search methods for identification of studies. 

This systematic review was performed according to the updated Preferred Reporting Items for Systematic Reviews and MetaAnalyses (PRISMA) statement and was registered in the International Prospective Register of Systematic Reviews (PROSPERO registration number: CRD42023406731) in 2023. A computer-assisted search in EMBASE and PubMed was carried out covering the years 1978–2022. Search terms used were [‘inflammatory bowel diseases’ OR ‘colitis ulcerative’ OR ‘Crohn’s disease’] AND [thrombosis]. No limits were imposed and the search was last updated on 25 October 2022. The bibliography of the selected articles was also screened to broaden the literature search.

Criteria for including studies. Types of studies: Observational studies, case series and case reports that met the variables of interest, written in English, French or Spanish. Types of participants: Adult or paediatric IBD patients diagnosed with SVT. 

Criteria for excluding studies. No data available to analyse for the observational study. Editorials, literature reviews, systematic reviews, meta-analyses and comments were retrieved. 

### 2.5. Data Collection and Analysis

All titles identified by the search were independently assessed by two reviewers (MP and HM) based on the title and abstract. Full texts of articles that did not provide the location of the thrombosis in the abstract were reviewed and removed from the selection. The eligible articles were assessed by examining the full-text paper and articles in which no features were described individually for the IBD patients with SVT were removed from the final analysis. Duplicates were identified and removed. In the case of a discrepancy between the two reviewers, a third reviewer (ED) solved the differences. Data were extracted and stored in an Excel Spreadsheet. Categorical variables are expressed as absolute values and frequencies, and continuous variables are expressed as the median and IQR for non-parametric variables.

## 3. Results

### 3.1. ENEIDA Cohort

Characteristics of inflammatory bowel disease in patients with splanchnic vein thrombosis.

Of the 59,000 patients included in the ENEIDA registry by August 2019, 133 patients had a diagnosis of venous thrombosis of an unspecified location or of the splanchnic area. Of these patients, 54 were excluded because of a thrombosis location other than splanchnic after checking out the medical records. Of the remaining 79 SVT cases, 30 were excluded from the final analysis due to missing mandatory variables, but they were also considered for the assessment of SVT prevalence. Finally, 49 cases of SVT from 25 centres were included. 

Table 1 summarises the demographic and IBD-related characteristics of the patients of the ENEIDA cohort. There was a predominance of men (69%) and the median age at diagnosis of SVT was 42 years (IQR, 34–49). There was also a predominance of Crohn’s disease (71%) with ileal involvement (80%) and an inflammatory or penetrating disease pattern. Most cases of ulcerative colitis had extensive disease. SVT occurred before IBD diagnosis in only three cases, while in one additional case, SVT was diagnosed at the same time as IBD. The most frequent clinical presentations of SVT were abdominal pain with or without fever (61%) and a radiological finding in the setting of active IBD (26%). Table 2 summarises the main clinical features of IBD at SVT diagnosis. At that time, most of the patients had active IBD as defined by a partial Mayo score > 2 or a Harvey–Bradshaw index > 6. In addition, more than half of the patients were undergoing treatment with immunosuppressants or biological agents, and/or were receiving systemic corticosteroids. Of note, only 22% of patients were receiving prophylaxis with low-molecular-weight heparin (LMWH) at the time of SVT.

Features and outcomes of splanchnic vein thrombosis episodes among inflammatory bowel disease patients.

The diagnosis of SVT was established based on the findings of abdominal CTs in 82% of the cases, whereas the remaining cases were diagnosed by abdominal ultrasound (10%) or, less often, MRI enterography (4%). The most frequently involved venous segments were the intrahepatic portal branches, the superior mesenteric vein, and the main trunk of the portal vein. Table 3 summarises the main characteristics of the cases of SVT. Of note, cavernomatous transformation of the portal vein was found in 16% of the patients at the time of SVT diagnosis.

Anticoagulation therapy was prescribed in 94% of the episodes (74% within the first month since diagnosis) and LWMH was the most frequently used medication (82%). Treatment was maintained for a median time of seven months. In 90% of the cases, a further radiological assessment of the splanchnic veins was available after a median of five months from the beginning of anticoagulation therapy. Gastroscopy was performed on only 16% of the patients after a median of five months (IQR 0.25–39) from SVT diagnosis. Although oesophageal varices were observed in 62% of these patients, no episodes of acute oesophageal variceal bleeding were recorded. 

Resolution or improvement of the SVT was observed in 61% and 16% of the cases, respectively, whereas thrombosis persistence without significant changes was observed in 15% and progression of the SVT in 7% of cases. No deaths were registered during the thrombotic episode.

Conditions associated with splanchnic vein thrombosis among inflammatory bowel disease patients.

**Figure 1:**
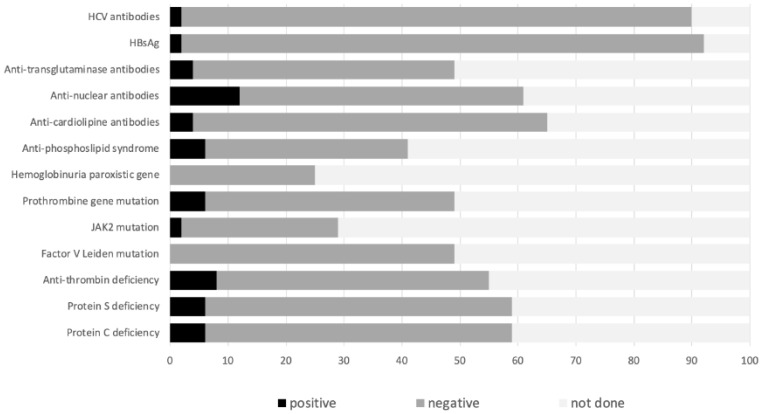
Expanded study of prothrombotic factors in the ENEIDA cohort.

Thirty-seven percent of the patients had at least one intraabdominal IBD-related complication (in addition to IBD itself) at SVT diagnosis, the most frequent being abscess and intestinal perforation (18% and 16%, respectively), followed by intestinal occlusion (12%) and toxic megacolon (2). In contrast, non-IBD-related inflammatory intraabdominal conditions accounted for a low proportion of cases (pancreatitis in 2%). Finally, 16% of the patients had a past history of venous thrombotic events, and eight patients had known chronic liver disease (primary sclerosing cholangitis in six, autoimmune liver cirrhosis in two)**.** A minimum expanded aetiologic work-up (arbitrarily defined by the determination of at least C and S proteins, anti-nuclear antibody, anti-transglutaminase antibody and hepatitis virus B and C serologies) was performed in 45% of patients. Figure 1 shows the screening performed for systemic prothrombotic conditions and the proportion of altered results. Table 4 shows the associated prothrombotic conditions found. In 27% of cases, at least one systemic (and non-related to IBD) prothrombotic condition was observed. This figure increased to 37.5% in the eight patients who had a previous thrombotic event. The systemic prothrombotic condition observed most frequently was antithrombin deficiency (8%), followed by prothrombin gene G20210A mutation, and C or S protein deficiency (6% each). Anti-nuclear antibodies were positive in six patients, although two of them had been previously exposed to anti-TNF agents. Coeliac disease was finally diagnosed in three patients following a positive serologic screening.

### 3.2. Systematic Review

**Figure 2:**
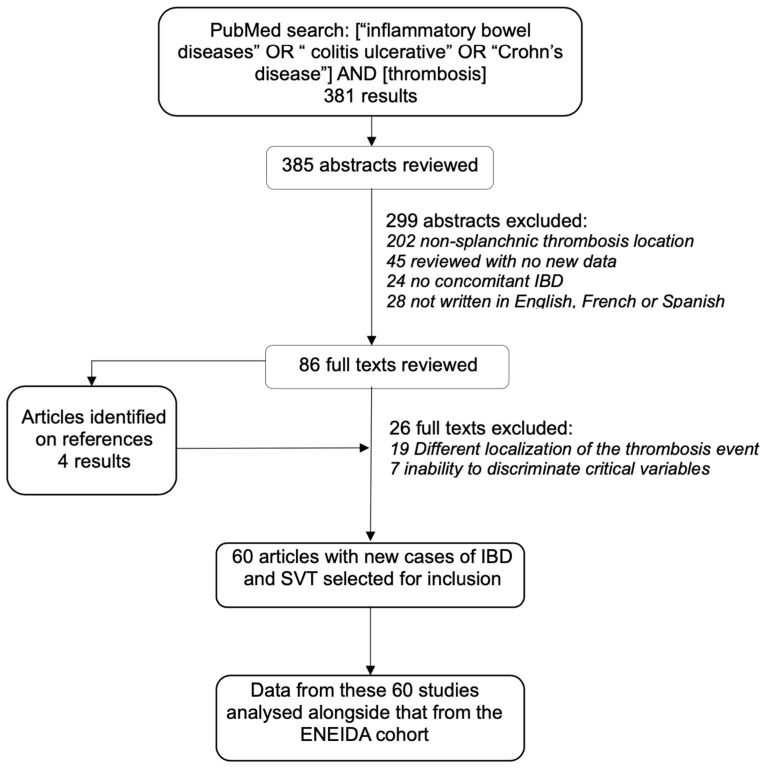
Flow diagram of study selection process for systematic review.

A total of 385 abstracts were identified from the literature search. Eighty-six full-text articles were reviewed and sixty were selected for inclusion in the analysis (Figure 2). The oldest article was published in 1979 and the most recent one in 2022; 44 out of 60 were published after 2000. All of them were retrospective, two were multicentre studies [4,11], six were single-centre studies [19,20,21,22,23,24] and fifty-two were case reports or case series [25,26,27,28,29,30,31,32,33,34,35,36,37,38,39,40,41,42,43,44,45,46,47,48,49,50,51,52,53,54,55,56,57,58,59,60,61,62,63,64,65,66,67,68,69,70,71,72,73]. Most of the articles reported between one and five cases of SVT, and only four studies reported more than thirty [4,11,19,24]. Of note, one of the multicentre studies and three case series only included thromboses of the superior mesenteric or portal veins.

A total of 318 patients with IBD and SVT were identified from the systematic review. Available demographic and IBD-related features of individual cases found in the systematic review are summarised in Table 1. Although the median age was younger than in the ENEIDA cohort, the median age in cohort studies [11,19] was quite similar to ours. Contrarily to our cohort, cases in the systematic review were more evenly distributed among CD (52%) and UC (48%) and SVT was diagnosed after surgery in a large proportion of cases (50%). Inflammatory disease activity was qualitatively available in only 173 patients (54%), the disease being active in 57% of them at the time of SVT diagnosis (Table 2). Moreover, in 26 out of 149 (18%) of the patients, an IBD-related intraabdominal inflammatory complication was present at that time, being abscess (*n* = 26), fistula (*n* = 8) or perforation (*n* = 2). Additionally, 53 out of 108 (49%) presented other non-IBD-related inflammatory conditions: systemic infection in forty-seven, hepatic abscess in four and acute pancreatitis in one. Three patients had abdominal malignancies (colonic adenocarcinoma in two and pancreatic cancer in one).

As observed in the ENEIDA cohort, the most frequent clinical presentation was abdominal pain (52%) followed by the incidental finding in cross-sectional imaging techniques (27%); of note, new onset of ascites accounted for 9% of cases (Table 3). 

The type of diagnostic examinations and the location of SVT were available in most of the cases (Table 3). In line with the ENEIDA cohort, abdominal CT was the most frequent diagnostic procedure. Conversely, the most frequent SVT location was the main portal trunk in two-thirds of the cases, followed by the superior mesenteric vein, with only 8% involving only the intrahepatic portal branches.

Data on SVT-related therapy was available in 250 patients. Anticoagulation therapy was prescribed in 88% of patients, dicumarinics being the most frequently used drugs (60%), followed by LMWH (25%) and direct oral anticoagulants (15%). Two patients were treated by thrombolysis and radiologic thrombectomy was performed in one patient. The remaining 27 patients (11%) were not specifically treated for SVT. 

Data on thrombophilic work-up was available in 140 patients (44%). At least one abnormal finding was found in 20% of them. The most commonly reported was homocystinaemia (12%), followed by anti-phospholipid syndrome (4%), mutations in Factor V Leiden (3%) and prothrombin (3%) genes, JAK2V617F mutation (1%), and antithrombin III (1%) and protein S (1%) deficiencies.

## 4. Discussion

The incidence of SVT among IBD patients is unknown. To date, no prospective studies searching intentionally for the presence of SVT among patients with active IBD have been performed and most of them reported a low number of cases. In the new ENEIDA cohort of SVT, we found an estimated prevalence of 0.13%, a figure close to that observed in some single-centre studies [5,63], though remarkably lower than the 2.4% observed in the multicentre study by Landman et al. [11]. This low prevalence may be explained, at least in part, by the large proportion of patients in whom SVT symptoms can be confused with those of IBD itself, thereby complicating a clinical suspicion of SVT. In fact, one-quarter of cases in both the ENEIDA cohort and the systematic review were diagnosed as incidental (not suspected) findings in an abdominal CT. Moreover, SVT can also be asymptomatic, and this may also explain the fact that 6% to 16% of the patients presented cavernous transformation of the thrombosis at diagnosis. 

Multiple factors have been associated with an increased risk of thrombotic events in IBD, particularly inflammatory disease activity ([7,8,9]). In our cohort, disease activity was measured objectively with clinical scores and, in line with previous studies, we found that SVT occurred in patients with active IBD in two-thirds of the cases [11,20]. Although individual data from the systematic review were available only qualitatively and in 173 patients, more than half of them also had active disease. In contrast, some studies have suggested that most SVTs occur in cases of inactive IBD [5,74]. However, despite the increasing use of CT and MRI enterography for disease monitoring among IBD patients over recent decades, no red flags regarding the risk of non-symptomatic SVT have been raised. Surprisingly (particularly when considering that most of the cases occurred concomitantly with active disease), less than one-fifth of patients in our cohort and also in the systematic review were receiving prophylaxis with LMWH, highlighting the need for thromboprophylaxis in patients with active inflammatory activity, as recommended in current guidelines [17,75].

In addition, and keeping in mind that there are many well-established conditions associated with SVT beyond IBD itself, particularly in non-cirrhotic patients, aetiologic studies for additional prothrombotic conditions are not frequently performed. As observed in both the ENEIDA cohort and the systematic review, these aetiologic studies were expanded in less than half of the patients. In spite of this, additional systemic prothrombotic conditions were identified in one-fifth to one-fourth of the patients in the systematic review and the ENEIDA cohort, respectively. This figure is slightly lower than that described by Landman et al., who observed a prothrombotic disorder in one-third of their cases [11]. It is difficult to draw reliable conclusions regarding the role of these coexisting prothrombotic conditions in the setting of IBD due to the heterogenicity of patient populations and the additional studies for prothrombotic conditions performed in the available literature but it seems reasonable to expand an aetiologic study given the proportion of patients with additional systemic risk factors for thrombotic events, although some authors suggest limiting these studies to patients in whom SVT occurs during inactive disease and no recent surgery or a past history of other thromboembolic events [19]. A better knowledge of the impact of coexisting prothrombotic conditions might clarify the need for long-term anticoagulation or an expanded indication for thromboprophylaxis in these patients. 

Regarding diagnostic issues, although this issue could not be assessed in the systematic review, another relevant finding was the low proportion of patients on whom a gastroscopy was performed. Interestingly, oesophageal varices were present in more than half of them. Although no patient developed variceal bleeding, this is a severe portal hypertension complication associated with a high mortality rate. Therefore, ruling out the development of oesophageal varices seems to be warranted.

Anticoagulation was prescribed in almost all the patients. In the ENEIDA cohort, neither major complications of anticoagulation therapy nor deaths were registered. Moreover, improvement or the complete resolution of the SVT took place in two-thirds of the patients. Although these excellent outcomes cannot be attributed unequivocally to anticoagulation, anticoagulation therapy for SVT in IBD patients seems to be both effective and safe. In fact, in the largest cohort of IBD-related SVT (limited to portal vein thrombosis with or without extension to other vessels) published to date, complete radiologic resolution was observed in 71% of patients. The authors stated that this was clearly superior to that reported in studies of all portal vein thrombosis and attributed this high resolution rate to some intrinsically benign property of IBD-related SVT (as non-IBD-specific cohorts may be enriched with patients who have SVT related to underlying prothrombotic disorders) or the high rates and prompt initiation of anticoagulation in IBD patients [19].

Anticoagulation therapy differed between the ENEIDA cohort and data in the systematic review, and this difference may be explained by the use of different treatment algorithms regarding geography and also because the reported cases occurred during a wide period of time. In our opinion, although both LMWH and vitamin K antagonists are considered valid therapeutic options [76,77], LMWH should be the drug of choice over oral treatments given the possibility of intestinal absorption abnormalities and drug interactions with IBD-related drugs. 

In conclusion, SVT does not seem to be a frequent event among patients with IBD and most cases occur in active disease without thromboprophylaxis, often with intraabdominal disease-related complications or soon after surgery. It must be borne in mind that SVT may mimic IBD-related symptoms or may occur silently but may cause pre-hepatic portal hypertension. However, IBD-related SVT is usually solved with anticoagulation therapy. Finally, even in the absence of solid evidence, additional studies for prothrombotic conditions are advised, at least in patients with a past history of other thromboembolic events or with SVT occurring in inactive IBD, given that at least one-fifth of patients share coexisting systemic prothrombotic conditions and this may change anticoagulation regimens.

## Figures and Tables

**Table 1:** Demographic and inflammatory bowel disease-related characteristics of the patients with splanchnic venous thrombosis.

	ENEIDA Cohort (*n* = 49)	Systematic Review (*n* = 318) *(available cases)*
Age (years) *	42 (34–49)	34 (25–44) *(78)*
Male gender	34 (69)	163 (51) *(318)*
Ethnicity *Caucasian/Black*	48 (98)/1 (2)	14 (74)/0 (0) *(19)*
Active alcoholism	4 (8)	*None available*
Active smoking	10 (20)	56 (30) *(189)*
Crohn’s disease/Ulcerative colitis	35/14 (71/29)	164/153 (52/48) *(317)*
Ulcerative colitis extent *proctitis/left-sided/extensive*	1/1/12 (7/7/86)	0/12/69 (0/14/85) *(81)*
Crohn’s disease location *Ileal/colic/ileocolic/upper gastrointestinal*	12/3/17/4 (33/8/47/8)	13/24/11 (27/50/23) (*48*)
Crohn’s disease behaviour *inflammatory/structuring/penetrating*	13/8/13 (37/23/37)	*None available*
Perianal disease	10 (22)	11 (18) *(63)*
Extraintestinal manifestations	20 (41)	3 (13) (*24*)

* median was calculated from the articles in which individual data were provided; in some cohort studies, age was provided as a median and ranged from 41 (19–69) [11] to 42 (29–55) [19] years.

**Table 2:** Main characteristics of inflammatory bowel disease at splanchnic venous thrombosis diagnosis. IBD = inflammatory bowel disease; LMWH = low-molecular-weight heparin; SVT = splanchnic venous thrombosis; NA = none available.

	ENEIDA Cohort (*n* = 49)	Systematic Review (*n* = 318) (*available cases*)
Active IBD	25 (68)	98 (57) *(173)*
Partial Mayo score (ulcerative colitis)	2 (0–5)	NA
Harvey–Bradshaw index (Crohn’s disease)	8 (6–10)	NA
Time since IBD diagnosis *(months)*	106 (7–189)	72 (2–162) (*49)*
IBD treatments at SVT diagnosis		
None	19 (39)	17 (9)
Immunosuppressants	15 (31)	53 (29)
Biological agents	12 (25)	77 (42)
Systemic corticosteroids	12 (25)	107 (59) *(183)*
LMWH thromboprophylaxis	11 (22)	16 (24) *(66)*
C-reactive protein (mg/L)	25 (14–64)	NA

**Table 3:** Main characteristics of splanchnic venous thrombosis (ENEIDA cohort and systematic review). SVT = splanchnic venous thrombosis; CT scan = computed tomography scan; MRI = magnetic resonance imaging.

	ENEIDA Cohort (n = 49)	Systematic Review (n = 318) (available cases)
Clinical presentation		237 (75)
Abdominal pain	20 (45)	125 (52)
Incidental finding	13 (26)	65 (27)
Ascites	0 (0)	4 (9)
Abdominal pain and fever	8 (16)	0 (0)
Fever	3 (6)	18 (8)
Nausea and vomiting	0 (0)	5 (2)
Variceal bleeding	0 (0)	2 (1)
Upper gastrointestinal bleeding	2 (4)	0 (0)
Intestinal ischemia	1 (2)	0 (0)
Location of thrombosis		238 (75)
Intrahepatic portal vein	25 (51)	18 (8)
Superior mesenteric vein	23 (47)	71 (30)
Main portal trunk	18 (37)	160 (67)
Splenic vein	5 (11)	34 (14)
Inferior mesenteric vein	1 (2)	3 (1)
Suprahepatic vein	1 (2)	2 (1)
Cavernomatous transformation at SVT diagnosis	8 (16)	14 (6)
Radiological examinations performed		241 (76)
Abdominal CT scan	40 (82)	214 (89)
Abdominal ultrasonography	19 (39)	36 (15)
MRI enterography	4 (8)	26 (11)

**Table 4:** Associated prothrombotic conditions found in the ENEIDA cohort.

Previous abdominal surgery	12 (25)
Digestive malignancies	1 (2)
Extraintestinal malignancies	7 (14)
Abdominal inflammatory disorder	6 (12)
Immunological disorders	2 (4)
Variable common immunodeficiency	1 (2)
Haemolytic anaemia	1 (2)
Haematological disorders	6 (12)
Purpura idiopathic thrombocytopenic	1 (2)
Other haematological disorders *	5 (10)
Previous thrombotic episodes	8 (16)
Deep thrombosis EEII	6 (12)
Pulmonary thromboembolism	0
Thrombosis in other locations **	2 (4)
Previous pregnancy	6 (12)
Previous miscarriage	1 (2)
IBD-related intraabdominal inflammatory conditions	
Abscess	9 (18)
Intestinal occlusion	8 (16)
Intestinal perforation	6 (12)
Megacolon	1 (2)
Non-IBD-related intraabdominal inflammatory conditions	
Acute pancreatitis	1 (2)

* 1 plasmocytoma, 1 primary cerebral lymphoma, 1 fungoid mycosis, 1 thrombocytosis, 1 lymphocytosis; ** 1 femoro-iliac and 1 haemorrhoidal thrombosis.

## Data Availability

The data underlying this article will be shared on reasonable request to the corresponding author.

## Abbreviations

CT	Computed tomography
IBD	Inflammatory bowel disease
LMWH	Low-molecular-weight heparin
MRI	Magnetic resonance imaging
SVT	Splanchnic venous thrombosis

## Appendix A. Complete Affiliations of the ENEIDA-GETECCU Investigators

Luis Bujanda, Hospital de Donostia (Donostia); Margalida Calafat, Hospital Germans Trias i Pujol (Badalona), Centro de Investigación Biomédica en Red de Enfermedades Hepáticas y Digestivas (Madrid); Xavier Calvet, Hospital Universitari Parc Taulí (Sabadell), Universitat Autònoma de Barcelona, (Barcelona), Centro de Investigación Biomédica en Red de Enfermedades Hepáticas y Digestivas (Madrid); Fiorella Cañete, Hospital Germans Trias i Pujol, (Badalona), Centro de Investigación Biomédica en Red de Enfermedades Hepáticas y Digestivas (Madrid); Jesús Castro, Hospital Clínic i Provincial de Barcelona (Barcelona); Ruth de Francisco, Hospital Central de Asturias (Oviedo); Eugeni Domènech, Hospital Germans Trias i Pujol, (Badalona), Centro de Investigación Biomédica en Red de Enfermedades Hepáticas y Digestivas (Madrid), Universitat Autònoma de Barcelona; Francisco Javier García Alonso, Hospital Río Hortega (Valladolid); María José García, Hospital Marqués de Valdecilla (Santander); Javier P. Gisbert, Hospital Universitario de La Princesa, Instituto de Investigación Sanitaria Princesa (IIS-IP), Universidad Autónoma de Madrid, Centro de Investigación Biomédica en Red de Enfermedades Hepáticas y Digestivas (Madrid); Fernando Gomollón, Hospital Clínico Lozano Blesa, IIS Aragón (Zaragoza), Centro de Investigación Biomédica en Red de Enfermedades Hepáticas y Digestivas (Madrid); Carlos González-Muñoza, Hospital de la Santa Creu i Sant Pau (Barcelona); Irene González Partida, Hospital Puerta de Hierro (Madrid); José M Huguet, Hospital General Univeristari de València (Valencia); Marisa Iborra, Hospital Politècnic i Universitari La Fe (Valencia); López Serrano, Hospital Universitario Fundación de Alcorcón (Alcorcón); Míriam Mañosa, Hospital Germans Trias i Pujol (Badalona), Centro de Investigación Biomédica en Red de Enfermedades Hepáticas y Digestivas (Madrid); María Dolores Martín-Arranz, Hospital La Paz (Madrid), Innate Immunity Group, IdiPAZ Institute for Health Research La Paz Hospital, (Madrid); Helena Masnou, Hospital Germans Trias i Pujol (Badalona), Centro de Investigación Biomédica en Red de Enfermedades Hepáticas y Digestivas (Madrid); Luis Menchén, Hospital Gregorio Marañon (Madrid), Centro de Investigación Biomédica en Red de Enfermedades Hepáticas y Digestivas (Madrid); Francisco Mesonero, Hospital Universitario Ramón y Cajal (Madrid); Estefanía Pérez-Rabasco, Hospital del Elche (Alincante); Maria Puig, Hospital Germans Trias i Pujol (Badalona); Empar Sáinz, Althaia Xarxa Assistencial Universitària de Manresa (Manresa); Sierra, Hospital de León (León); Gerard Surís Hospital Universitari de Bellvitge, IDIBELL, (L’Hospitalet del Llobregat), Carlos Taxonera, Hospital Clínico San Carlos (Madrid); Raquel Vicente, Hospital Miquel Servet (Zaragoza).
